# Inhibition of the Maillard Reaction by Phytochemicals Composing an Aqueous Coffee Silverskin Extract via a Mixed Mechanism of Action

**DOI:** 10.3390/foods8100438

**Published:** 2019-09-25

**Authors:** Miguel Rebollo-Hernanz, Beatriz Fernández-Gómez, Miguel Herrero, Yolanda Aguilera, María A. Martín-Cabrejas, Jaime Uribarri, María Dolores del Castillo

**Affiliations:** 1 Institute of Food Science Research (CIAL, UAM-CSIC), C/Nicolás Cabrera, 9, Universidad Autónoma de Madrid, 28049 Madrid, Spain; miguel.rebollo@uam.es (M.R.-H.); b.fernandez@csic.es (B.F.-G.); m.herrero@csic.es (M.H.); marí; 2 Department of Agricultural Chemistry and Food Science, Faculty of Science, C/Francisco Tomás y Valiente, 7, Universidad Autónoma de Madrid, 28049 Madrid, Spain; 3 Department of Medicine, The Icahn School of Medicine at Mount Sinai, 1468 Madison Ave, New York, NY 10029, USA; jaime.uribarri@mountsinai.org

**Keywords:** advanced glycation end products (AGE), antioxidants, chlorogenic acid, coffee silverskin extract, genistein, melatonin, protein glycation, protein glycoxidation, Maillard reaction

## Abstract

This work aimed to evaluate the contribution of isoflavones and melatonin to the aqueous extract obtained from the coffee silverskin (CSE) antiglycative properties, which has not been previously studied. To achieve this goal, two model systems constituted by bovine serum albumin (BSA) and reactive carbonyls (glucose or methylglyoxal) in the presence or absence of pure phytochemicals (chlorogenic acid (CGA), genistein, and melatonin) and CSE were employed. Glucose was used to evaluate the effect on the formation of glycation products formed mainly in the early stage of the reaction, while methylglyoxal was employed for looking at the formation of advanced products of the reaction, also called methylglyoxal-derivative advanced glycation end products (AGE) or glycoxidation products. CGA inhibited the formation of fructosamine, while genistein and melatonin inhibited the formation of advanced glycation end products and protein glycoxidation. It was also observed that phenolic compounds from CSE inhibited protein glycation and glycoxidation by forming BSA–phytochemical complexes. CSE showed a significant antiglycative effect (*p* < 0.05). Variations in the UV-Vis spectrum and the antioxidant capacity of protein fractions suggested the formation of protein–phytochemical complexes. Fluorescence quenching and *in silico* analysis supported the formation of antioxidant–protein complexes. For the first time, we illustrate that isoflavones and melatonin may contribute to the antiglycative/antiglycoxidative properties associated with CSE. CGA, isoflavones, and melatonin composing CSE seem to act simultaneously by different mechanisms of action.

## 1. Introduction

The Maillard reaction results from the reaction between a reactive carbonyl group of reducing sugars and the free amino groups of proteins, without the participation of enzymes, giving rise to Amadori products. α-Dicarbonyls, such as glyoxal (GO), methylglyoxal (MGO), and deoxyosones, are reactive intermediate species able to accelerate the protein glycation reaction due to their higher reactivity compared to glucose. Oxidative (glycoxidation) and non-oxidative subsequent reactions result in the formation of a variety of advanced glycation end products (AGE) [1].

AGEs are formed continuously in the human body at a slow rate, but this rate is increased by hyperglycaemia and oxidative stress status [2]. Moreover, research has shown that dietary AGEs, indistinguishable from endogenous AGEs, are important contributors to the body AGE pool [3]. Dietary AGEs are at least partially absorbed from the gut and interact systemically with the AGE receptor (RAGE) [4]. AGEs might also form in the gut during food digestion [5]. AGEs have been associated with oxidative stress and inflammation, underlying abnormalities behind most non-infectious chronic diseases, including cardiovascular disease, diabetes, chronic kidney disease, and neurodegenerative diseases [6]. 

The inhibition of the Maillard reaction *in vivo* has been obtained through different strategies over the years [7]. Finding inhibitors of the Maillard reaction in foods and *in vivo* is still an ongoing quest. Counteracting the Maillard reaction by the addition of natural extracts and phytochemicals has also been targeted, as well as the removal or modification of one of the reactants (the amino groups or the reducing sugars) and the trapping of α-dicarbonyl compounds. Some phenolic compounds have also been proposed to react with amines to form either benzoquinone imines or amine–quinone adducts via a Michael addition, thus avoiding the progress of the reaction [8,9]. Overall, antiglycative mechanisms comprise any mechanisms that delay the Maillard and subsequent glycoxidation reactions [10], also including the scavenging of hydroxyl and superoxide radicals, the reduction of the generation of reactive carbonyl or dicarbonyl groups, and the chelation of metal ions promoting oxidative reactions.

Hence, the search for a natural product with inhibitory potential against the Maillard reaction is a challenging strategy to evaluate food products containing phytochemicals of interest. Coffee by-products have been proposed as food ingredients loaded with bioactive compounds that are safe to use [11,12]. Coffee silverskin, the outer layer of green coffee beans and a by-product of the roasting procedure, is a good source of phenolic compounds, such as chlorogenic acid (CGA) [13]. The inhibitory effect of CGA and coffee silverskin is associated with its carbonyl trapping capacity as well as its ability to react with side-chains of protein amino residues blocking the reaction sites [14]. Nevertheless, the overall antiglycative capacity of coffee silverskin could also be associated with phytochemicals other than CGA, such as isoflavones and alkaloids, and the mechanism of action of these other compounds from coffee silverskin is still not clearly understood. Genistein has been shown to inhibit the formation of AGE by forming adducts with MGO both *in vitro* and *in vivo* [15,16]. Other isoflavones also inhibit glycation [17]. Likewise, melatonin could present antiglycative properties [18]. However, no studies have described the presence of these compounds in coffee silverskin extracts nor their contribution to the antiglycative effect attributed to them. 

We hypothesised that different phytochemical compounds from coffee silverskin, besides CGA, could provide antiglycative effects through different mechanisms. Hence, the present study aimed to identify other potential contributors to the antiglycative properties of coffee silverskin extract, besides CGA, by analysing the effects of a coffee silverskin aqueous extract and other pure compounds present therein, to assess their inhibitory effects against the Maillard reaction and their mechanisms *in vitro* and *in silico*.

## 2. Materials and Methods 

### 2.1. Materials

All reagents and solvents used were of analytical grade. Bovine serum albumin (BSA), glucose, aminoguanidine, CGA, genistein, melatonin, caffeine, rutin, sodium azide, *O*-phthaldehyde (OPA), *N*_α_-acetyl-l-lysine, 1-deoxy-1-morfolinofructose (DMF), nitroblue tetrazolium (NBT), *o*-phenylenediamine (OPD), trichloroacetic acid (TCA), 2,4-dinitrophenylhydrazine (DNPH), guanidine, and the Folin–Ciocalteu reagent were supplied by Sigma-Aldrich (St. Louis, MO USA). Other chemicals and their suppliers were as follows: β-mercaptoethanol (Merck, Hohenbrunn, Germany), MGO and diammonium salt-2,2´-azino bis (3-ethylbenzthiazoline-6-sulfonic acid) (ABTS) from Fluka, (Buchs, Switzerland), and Bradford reagent from Bio-Rad (München, Germany). The Amicon^®^ Ultra 0.5 mL centrifugal filter unit fitted with an Ultracel^®^ 30 K regenerated cellulose membrane (30 kDa cut-off) was from Merck Millipore Ltd. (Cork, Ireland). Microtest 96-well polystyrene plates made from high-quality polystyrene were purchased from Sarstedt AG & Co. (Nümbrecht, Germany). The AGE determination kit was supplied by Lamider^®^ (Mexico D.F., Mexico). The Milli-Q water used was obtained using a purification system (Millipore, Molsheim, France).

### 2.2. Coffee Silverskin Extract (CSE) Preparation

Coffee silverskin from Arabica (*Coffea arabica*) and Robusta (*Coffea canephora*) species was provided by Qualery Culture S.L. (Val de Santo Domingo, Spain). CSE was prepared by aqueous extraction according to a patented procedure [19]. Briefly, 50 mL of boiling water was added to 2.5 g of coffee silverskin (Arabica: Robusta, 1:1, *w*/*w*) and the mixture was maintained in continuous stirring for 10 min. The mixture was filtered through Whatman qualitative filter paper (Whatman, Mainstone, UK), grade 4, and was freeze-dried. The powdered extracts were stored in dark and dry conditions until further analysis. 

### 2.3. Phytochemical Characterisation of CSE

#### 2.3.1. Total Phenolic Compounds (TPC)

TPCs were determined by the Folin–Ciocalteu colourimetric method [20]. The reduction reaction was carried out in 210 μL total volume in 96-well microplates. Ten μL of the sample was added to 150 μL of Folin–Ciocalteu reagent (diluted 1:14, *v*/*v* in Milli-Q water). After exactly 3 min, 50 μL of Na_2_CO_3_ (20%, *w*/*v*) was added into each well. Absorbance was recorded at 750 nm using a microplate reader BioTek PowerWave™ XS (BioTek Instruments, Winooski, VT, USA). Calibration curves were constructed using standard solutions of CGA (0.1–1 mg L^−1^), and results were expressed as μg CGA equivalents mg^−1^ (μg CGAE mg^−1^). 

#### 2.3.2. Total Flavonoids Content (TFC)

TFC was quantified as previously described [21]. Briefly, 100 µL of CSE was mixed with 30 µL of 5% NaNO_2_ and samples were incubated at room temperature for 5 min. Subsequently, 30 µL of 10% AlCl_3_ was added and incubated for 6 min. Then, 100 µL of NaOH 2 mol L^−1^ was added, vortexed, and the absorbance was read at 510 nm. TFC was calculated with a calibration curve of rutin, and the results were expressed as µg rutin equivalents mg^−1^ (µg RE mg^−1^). 

#### 2.3.3. Melatonin Quantification by High-Performance Liquid Chromatography-Electrospray Ionization Tandem Mass Spectrometry (HPLC-ESI-MS/MS)

CSE was dissolved in Milli-Q water (10 mg mL^−1^) and passed through a 0.45 μm pore-size nylon membrane syringe filter (Análisis Vínicos, Ciudad Real, Spain). Melatonin isolation and quantification were performed according to published instructions [22]. Melatonin was determined by HPLC-ESI-MS/MS triple quadrupole (Varian 1200 L with Atmospheric Pressure Ionization-Electrospray Ionisation (API-ES) between 10 and 1500 Da range mass). An Intensity Trio C18 (150 mm × 2.0 mm ID, S-3 μm, 12 nm BRYTC18032150) (Bruker, Billerica, MA, USA) column and a flow rate of 0.2 mL min^−1^ were used. Acetonitrile containing 0.1% formic acid (solvent A) and 0.1% formic acid in H_2_O (solvent B) as mobile phase were pumped with the following gradient: 5% solvent A (0–4 min), 5–100% solvent A (4–15 min), 100% solvent A (15–18 min), and 100–5% solvent A (18–25 min) to recover initial conditions. Melatonin was recorded using multiple reaction monitoring (MRM) mode by selecting ion 233 ([M + H]^+^) at the first quadrupole (Q1), fragmented in Q2, and analysing the resulting ion *m/z* 130, 131, 159, and 174 at Q3, measuring at 174. Quantification was performed by the external standard method, using pure melatonin (Sigma-Aldrich Química, Madrid, Spain) dissolved in MeOH/H_2_O (80:20, *v*/*v*) containing 0.1% formic acid. Melatonin content was expressed as ng mg^−1^ CSE.

#### 2.3.4. CGA and Caffeine Quantification by Ultra-Performance Liquid Chromatography-Electrospray Ionization Tandem Mass Spectrometry (UPLC-ESI-MS/MS)

CGA and caffeine were determined by UPLC-ESI-MS/MS. CSE dissolved in Milli-Q water (10 mg mL^−1^) was filtered using a 0.45 μm pore-size nylon membrane syringe filter (Análisis Vínicos, Tomelloso, Spain). Aliquots (10 μL) were analysed using an Accela liquid chromatograph (Thermo Scientific, San Jose, CA, USA) coupled to a TSQ Quantum^TM^ (Thermo Scientific, San Jose, CA, USA) triple quadrupole analyser via an electrospray ionisation (ESI) interface. The mass spectrometer was operated in the positive ESI mode to quantify caffeine and in the negative ESI mode to quantify CGA. Two transition ions were monitored for identification, but only the most intense one for each precursor ion was used for quantification. Parent ([M – H]^‒^) and product ions for CGA were *m/z* 353.2 and 191.1, respectively, whereas parent ([M – H]^+^) and product ions for caffeine were *m/z* 195.1 and 138.2, respectively. CGA and caffeine content was expressed as µg mg^−1^ CSE.

#### 2.3.5. In Vitro Antioxidant Capacity 

The antioxidant capacity of samples was estimated by the ABTS^•+^ decolourisation assay, as previously described [23]. 2.2′-Azino-bis(3-ethylbenzothiazoline-6-sulfonic) acid radical cations (ABTS^•+^) were produced by reacting 7 mmol L^−1^ ABTS stock solution with 2.45 mmol L^−1^ potassium persulfate and allowing the mixture to stand in the dark at room temperature for 12–16 h before use. The ABTS^•+^ solution (stable for two days) was diluted in 5 mmol L^−1^ phosphate buffer (PBS) pH 7.4 to an absorbance of 0.70 ± 0.02 at 734 nm. Each sample was dissolved in PBS (5 mmol L^−1^, pH 7.4) at 0.1 mg L^−1^. Thirty μL of the test sample and 200 μL of diluted ABTS^•+^solution were mixed. The absorbance of the samples at 734 nm was measured at 10 min of reaction using a BioTek Power Wave™ XS microplate reader. CGA at concentrations of 0.015–0.2 mmol L^−1^ was used for calibration, and results were expressed as μg CGA equivalents mg^−1^ (μg CGAE mg^−1^).

### 2.4. Maillard Reaction In Vitro Models: Protein Glycation and Formation of Methylglyoxal-Derivate AGE

Two Maillard reaction model systems were prepared to investigate the effect of pure phytochemicals and CSE. The different model systems were employed for evaluating the effect of the phytochemicals on the glycation (BSA–glucose (BSA-GLU) model system) and glycoxidation (BSA–MGO) reactions. The former provided information on the effect of the phytochemicals on the different steps of the Maillard reaction, including Amadori rearrangement (early stage), while the latter provided information on the trapping of carbonyl groups and formation of advanced products of the Maillard reaction and protein oxidation products. Before the initiation of the glycation or glycoxidation reactions by the addition of glucose or MGO, the pH values of all solutions were measured at 25 °C using an electrode pH-meter (Metler Toledo, Madrid, Spain) to ensure optimal and equal conditions of reaction in all samples (pH = 7.4). All samples were prepared in triplicate. A control solution of BSA alone was also included. 

BSA, glucose, and MGO were dissolved in PBS (0.01 mol L^−1^, pH 7.4, 0.02 g mL^−1^ sodium azide). BSA was mixed with glucose and incubated at 37 °C for 21 days in the absence or presence of inhibitors (aminoguanidine, CSE, CGA, genistein, and melatonin). In parallel, inhibitors were incubated with BSA at 37 °C for 21 days to measure their intrinsic fluorescence. Likewise, BSA was mixed with MGO and incubated at 37 °C for 72 h. The final concentration of the reactants was 10 mg mL^−1^ BSA (150 µmol L^−1^), 100 mg mL^−1^ glucose, 5 mmol L^−1^ MGO, 5 mmol L^−1^ aminoguanidine, genistein, and melatonin and 1, 5, or 10 mmol L^−1^ CGA.

The progress of the protein glycation reaction was determined by analysing free amino groups, fructosamine, fluorescent and total AGE, and protein carbonyls. The progress of the formation of MGO-derivative AGE was determined by analysing free amino groups, fluorescent AGE, and protein carbonyls.

### 2.5. Assessment of the Degree of Progress of the Maillard Reaction 

#### 2.5.1. Free Amino Groups

Free protein amino groups (both *N*-terminal and epsilon –NH_2_ of lysine) were determined by the OPA assay. The OPA reagent was freshly prepared by dissolving 10 mg of OPA in 250 μL of 95% (*v*/*v*) ethanol and adding 9.8 mL of 0.01 mol L^−1^ PBS pH 7.4 and 20 μL of β-mercaptoethanol. The reaction mixture was composed of 10 μL of the sample, containing 1 mg mL^−1^ of protein, 100 μL of OPA reagent, and 140 μL of Milli-Q water. The reaction was carried out in a transparent polystyrene 96-well microtest plate (No. 82.1581). Fluorescence was measured after the addition of the OPA reagent on a microplate fluorescence reader Biotek Synergy™ HT (Biotek Instruments, Winooski, VT, USA) using 360 ± 40 nm and 460 ± 40 nm as excitation and emission wavelengths. Fluorescence was measured every 53 s for 15 min. Calibration curves were constructed using standard solutions of *N*_α_-acetyl-l-lysine (0.025–1 mmol L^−1^). All measurements were performed in triplicate, and data were expressed as μg *N*_α_-acetyl-l-lysine equivalent per mg of protein (μg mg^−1^ prot.).

#### 2.5.2. Early Products of the Maillard Reaction

The formation of Amadori compounds was measured by the NBT assay, as previously described [24]. Briefly, samples (25 μL) were added to sodium carbonate buffer (100 μL, 100 mmol L^−1^, pH 10.8) with NBT (0.25 mmol L^−1^). Microplates were incubated for 20 min at 37 °C and measured spectrophotometrically against control at 530 nm. The fructosamine analogue DMF was used as a standard. All measurements were performed in triplicate and expressed as μg DMF equivalents mg^−1^ protein (μg DMF eq. mg^−1^ prot.). 

#### 2.5.3. Fluorescent AGE 

AGE formation was monitored by fluorescence spectrophotometry using a Biotek microplate spectrophotometer set at 360 ± 40 nm and 460 ± 40 nm as excitation and emission wavelengths. All measurements were performed in triplicate and expressed as relative fluorescence units mg^−1^ protein (RFU mg^−1^ prot.). 

#### 2.5.4. Total AGE

The content of total AGE was analysed by Enzyme-Linked Immuno Sorbent Assay (ELISA) using a commercial kit, according to the manufacturer's instructions (Lamider, México). A total of 100 μL of standard or sample was pipetted to each well (coated with an AGE-sensitive primary antibody), and 50 μL of the conjugate (peroxidase bound secondary antibody, primary antibody sensitive) was added. After incubation at 25 °C for at least 30 min, the wells were washed three times with 300 μL of the provided buffer. Then, 100 μL of the substrate–chromogen solution (10 mg OPD, 100 μL H_2_O_2_ 3.7% *v*/*v*, 11 mL buffer) was re-incubated for 30 min (exactly). Finally, 20 μL of the stop solution (1 mol L^−1^ H_2_SO_4_) was added. Absorbance was read at 492 and 530 nm. All measurements were performed in triplicate. The calculations were performed using logistic transformation, and the results were expressed as relative fluorescence units mg^−1^ protein (RFU mg^−1^ prot.). 

#### 2.5.5. Protein Oxidation

Protein oxidation or glycoxidation was measured as carbonyl groups content according to an adapted method described previously [25]. A total of 50 μL of the sample was derivatised with four volumes (200 μL) of DNPH (10 mmol L^−1^ 2,4-dinitrophenylhydrazine in 2 mol L^−1^ HCl). In parallel, a blank of each sample was made along with each sample, using 200 μL of 2 mol L^−1^ HCl. The samples were kept in the dark and under gentle agitation (400 rpm) for 1 h. Once derivatised, the protein samples were precipitated with 250 μL of TCA (20% *m*/*v*) and kept on ice for 30 min. Then, samples were centrifuged (7000× *g*, 5 min) and pellets were washed with ethanol: ethyl acetate (50:50, *v*/*v*) at least three times, to eliminate the remaining and non-reacted DNPH, and re-dissolved in 6 mol L^−1^ guanidine (prepared in 50 mmol L^−1^ phosphate buffer, pH 2.5). Absorbance was measured at 360 nm. Carbonyl content was expressed as nmol mg^−1^ protein using an extinction coefficient of 22,000 nmol L^−1^ cm^−1^. Protein concentration was determined by the Bradford method. 

### 2.6. Evaluation of the Formation of Protein–Phytochemical Complexes

Before analysis, the protein fraction of samples was isolated by ultrafiltration. Samples (0.4 mL) were placed in the sample reservoir of an Amicon^®^ Ultra 0.5 mL centrifugal filter unit fitted with an Ultracel^®^ 30 K regenerated cellulose membrane (30 kDa cut-off) (Millipore Ltd., Tullagreen, Ireland) and centrifuged at 14,000× *g* for 40 min at room temperature. The retentates were recovered and diluted in PBS (0.4 mL). Protein concentration was determined by the Bradford micromethod. The high molecular weight (HMW) protein fraction was used for structural and functional characterisation.

#### 2.6.1. UV-Vis Spectra 

A Biotek microplate UV-Vis spectrophotometer equipped with UV KC junior software (Biotek) was used. The spectrum of the isolated protein fraction was measured at 200–790 nm using a quartz 96-well microplate. The results were expressed as absorbance units (AU).

#### 2.6.2. Antioxidant Capacity of the Protein–Phytochemical Complexes

The antioxidant capacity of samples’ protein fractions was estimated by the ABTS^•+^ decolourisation assay, as previously described in Section 2.3.5. 

#### 2.6.3. Fluorescence Spectra

Fluorescence spectra measurements of the protein solutions were obtained with a Tecan Infinite 200 Pro fluorometer (Männedorf, Switzerland). There was no significant absorption/emission from the added molecules at the excitation wavelength (250 nm). Therefore, the observed fluorescence signal (*F*) was identified as the fluorescence contributed only by the tryptophan residue of the protein. All the data reported here are the average of three independent measurements. The protein fluorescence quenching data in the presence of CGA, genistein, and melatonin (quenchers, Q) was analysed with the Stern–Volmer equation:

(1) $$\frac{F_{0}}{F}=1+K_{SV}\left[Q\right]=1+k_{q}\tau_{0}\left[Q\right],$$

where *F_0_* and *F* represent the protein fluorescence in the absence and presence of quencher at concentration [*Q*], respectively. *K_SV_* is the Stern–Volmer quenching constant, *k_q_* is the bimolecular quenching constant, and *τ_0_* is the intrinsic tryptophan fluorescence lifetime (∼5.78 × 10^‒9^ s) in the absence of quencher. For the static quenching interaction, when small molecules bind independently to a set of equivalent sites on a macromolecule, the binding constant (*K_a_*) and the number of binding sites (*n*) can be determined by the following equation:

(2) $$\log\frac{\left(F_{0}-F\right)}{F}=\log K_{a}+n\log\left[Q\right],$$

where, *F_0_*, *F*, and [*Q*] are the same as in the previous equation, *K_a_* is the binding constant, and *n* is the number of binding sites per BSA molecule.

#### 2.6.4. In Silico Prediction of Protein–Phytochemical Interaction

*In silico* studies were performed using molecular docking methods to investigate the interactions of glucose, MGO, CGA, genistein, and melatonin with the different domains of BSA. The 3D crystal structure of BSA (PDB ID: 3V03) was downloaded from the Research Collaboratory for Structural Bioinformatics (RCSB) Protein Data Bank website (http://www.rcsb.org/pdb/home/home.do). Search space dimensions, centre point, and flexible torsions were assigned with AutoDock Tools, and the docking calculations were carried out using AutoDock Vina according to previous studies [26,27,28]. Water molecules in the BSA structure were removed, hydrogen atoms were added, and partial charges were assigned to the macromolecule. The structure of BSA was divided into three domains, I, II, and III, each subdivided into two subdomains, A and B. Compound structures were retrieved from the PubChem Compound database (https://pubchem.ncbi.nlm.nih.gov/). One hundred different runs were performed for each ligand, and the pose with the highest binding affinity (lowest binding energy) was saved. Protein–ligand interactions and binding modes were visualised in the Discovery Studio 2017 R2 Client (Dassault Systèmes Biovia Corp^®^, San Diego, CA, USA). 

### 2.7. Statistical Analysis

Experiments were performed in triplicate. The analysis of each chemical indicator was also carried out in triplicate. Results were expressed as mean ± standard deviation (SD) (*n* = 3) and analysed by one-way analysis of variance (ANOVA) and post hoc Tukey test. Differences were considered significant at *p* < 0.05. Bivariate correlation analyses to study the associations between results from different experiments were performed. To analyse the similarities among the antiglycative potentials of the samples, principal component analysis (PCA) and hierarchical cluster analysis (dendrogram) were accomplished. The statistical analysis of the results was performed using SPSS 23.0 (IBM, Armonk, NY, USA). Multivariate analyses were carried out with XLSTAT 2016 for Microsoft Excel 2016 (Addinsoft, Paris, France). 

## 3. Results

### 3.1. Characterisation of CSE 

The compositions of phytochemicals and the overall antioxidant capacity of CSE are shown in Table 1. The presence of phenolic compounds, CGA, flavonoids, melatonin, and caffeine was detected. Phenolic compounds and caffeine are the most abundant compounds among those analysed in the extract. It is worth noting the presence of melatonin (2.0 ng mg^−1^) in CSE. CGA (35.1 µg mg^−1^) represents about 50% of the total phenolic compounds, but flavonoids, including isoflavones, also have a significant role (83.2 µg RE mg^−1^) on the phytochemical load of CSE. The extract presented antioxidant capacity in values corresponding to the expected contribution of the studied phytochemicals.

### 3.2. Inhibition of the Maillard Reaction

#### 3.2.1. Protein Glycation in the BSA-GLU Model System

**Figure 1:**
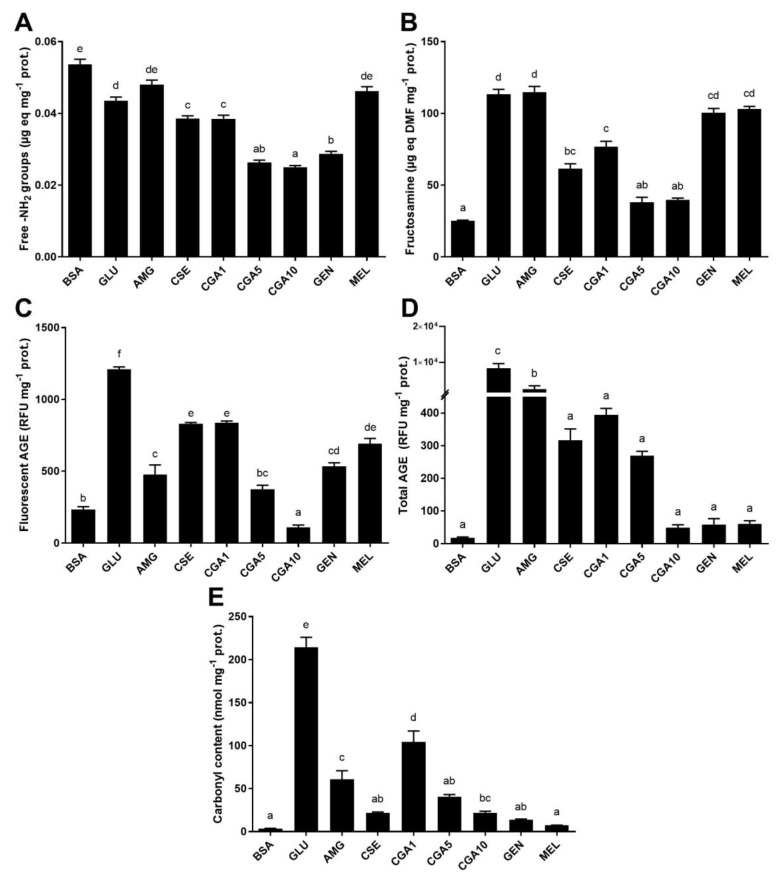
Contents of free amino groups (**A**), fructosamine (**B**), fluorescent AGE (**C**), total AGE (**D**), and protein oxidation (**E**) in the samples of the BSA-GLU glycation system (21 days incubation, 37 °C, pH 7.4). BSA: negative control; GLU: BSA + glucose; AMG: BSA + glucose + aminoguanidine; CSE: BSA + glucose + CSE (12,5 mg mL^−1^); CGA1: BSA + glucose + 1 mmol L^−1^ CGA; CGA5: BSA + glucose + 5 mmol L^−1^ CGA; CGA10: BSA + glucose + 10 mmol L^−1^ CGA; GEN: BSA + glucose + 5 mmol L^−1^ genistein; MEL: BSA + glucose + 5 mmol L^−1^ melatonin. Results are presented as mean ± SD (*n* = 3). Bars with different letters denote significant differences (*p* < 0.05) when subjected to the Tukey multiple range test.

Figure 1 shows the progress of the reaction between protein and glucose at pH 7.4, 37 °C for 21 days, in the presence and absence of phytochemicals. A significant (*p* < 0.05) loss of amino groups was observed in the control positive sample (GLU), which denoted the reaction (Figure 1A). CGA and genistein at concentrations higher than 5 mmol L^−1^ significantly decreased (*p* < 0.05) the content of free amino groups. CGA samples showed a dose–response effect in the content of amino groups being inversely proportional to CGA concentration (*r* = −0.976). CSE also produced a significant (*p* < 0.05) decrease in amino groups. Free amino groups’ values found in samples composed by aminoguanidine and melatonin were not significantly different (*p* > 0.05) to those detected in the control samples (BSA in the presence and absence of glucose). 

As expected, the formation of early Maillard reaction products, also called Amadori rearrangement compounds, was detected in the reaction mixture (Figure 1B). Their formation was significantly inhibited (*p* < 0.05) by the addition of CSE and CGA at high concentrations (5 and 10 mmol L^−1^). Aminoguanidine, genistein, and melatonin did not inhibit the early step of the Maillard reaction. 

Figure 1C shows the formation of fluorescent AGE. As can be observed, the addition of glucose produced a significant (*p* < 0.05) formation of fluorescent compounds. Their formation was significantly (*p* < 0.05) inhibited in the presence of aminoguanidine. CSE and 1 mmol L^−1^ CGA also significantly inhibited their formation (51 and 50%, respectively). A dose–response relationship between CGA concentration and fluorescent AGE formation was detected (*r* = ‒0.976). CGA, genistein, and melatonin at a concentration of 5 mmol L^−1^ showed effective inhibition of the formation of fluorescent compounds in the glycation mixtures. The inhibitory effect of genistein was comparable to that of aminoguanidine and melatonin (*p* > 0.05). Melatonin presented similar inhibition to that observed for 1 mmol L^−1^ CGA, genistein, and CSE (*p* > 0.05). 

Total AGE contents are shown in Figure 1D. A large formation of AGE was significantly inhibited by the addition of all the studied phytochemicals (95–100%, *p* < 0.05). As described for fluorescent AGE, a dose–response effect was noted for CGA (*r* = ‒0.996). Genistein, 10 mmol L^−1^ CGA, and melatonin were the most effective inhibitors of total AGE formation among the studied phytochemicals (*p* < 0.05). The addition of CSE in the glycation mixture significantly inhibited the formation of fluorescent compounds (*p* < 0.05).

Figure 1E shows the content of protein carbonyls due to oxidation during the glycation reaction. This formation was enhanced 600-fold in the presence of glucose. The addition of aminoguanidine, phytochemicals, and coffee by-product extract significantly inhibited (*p* < 0.05) the formation of carbonyls bound to BSA. CSE (92%), genistein (95%), and melatonin (98%) showed the highest capacity to inhibit the carbonyl content formation amount the studied samples. CGA (1 mmol L^−1^) had a lower effect for inhibiting the formation of protein carbonyls, followed by aminoguanidine. A dose–response inhibitory effect was observed for CGA. At higher concentrations (5 and 10 mmol L^−1^ CGA), the inhibition was similar (*p* < 0.05) to that of CSE, genistein, and melatonin.

**Figure 2:**
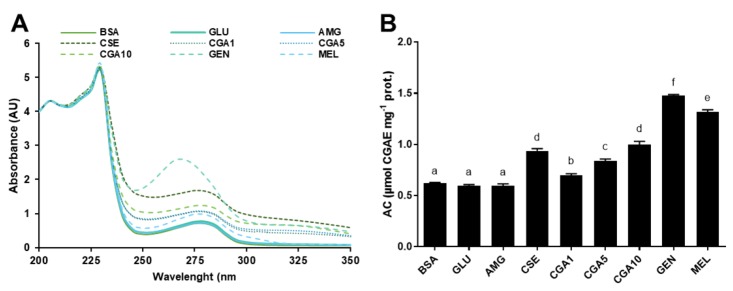
UV-Vis spectra (**A**) and antioxidant capacities (**B**) of the high molecular weight protein fractions (>30 kDa) isolated from the samples of the BSA-GLU glycation system (21 days incubation, 37 °C, pH 7.4) BSA: negative control; GLU: BSA + glucose; AMG: BSA + glucose + aminoguanidine; CSE: BSA + glucose + CSE (12,5 mg mL^−1^); CGA1: BSA + glucose + 1 mmol L^−1^ CGA; CGA5: BSA + glucose + 5 mmol L^−1^ CGA; CGA10: BSA + glucose + 10 mmol L^−1^ CGA; GEN: BSA + glucose + 5 mmol L^−1^ genistein; MEL: BSA + glucose + 5 mmol L^−1^ melatonin. Results are presented as mean ± SD (*n* = 3). Bars with different letters denote significant differences (*p* < 0.05) when subjected to Tukey multiple range test.

Figure 2 illustrates the modifications of the HMW protein fraction (>30 kDa) isolated after the incubation with glucose. The BSA UV-Vis spectrum (Figure 2A) was unaffected by the addition of glucose and aminoguanidine. However, the presence of CSE, CGA, genistein, and melatonin in the reaction mixture produced changes in the UV-Vis spectrum of the protein. An absorbance maximum was detected at 270–280 nm in all samples treated with phytochemicals. BSA antioxidant properties (Figure 2B) were not modified in the presence of glucose or aminoguanidine. However, the addition of pure phytochemicals and CSE resulted in the formation of protein–phytochemical complexes which possessed antioxidant properties. The antioxidant properties of the protein–phytochemical complexes (0.70–1.48 μmol CGAE mg^−1^ protein) were at least 12% higher than those corresponding to the native BSA. 

**Figure 3:**
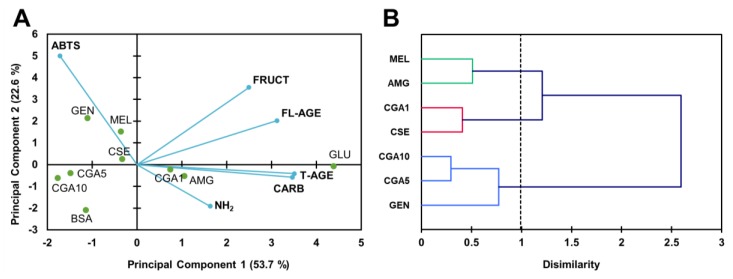
Biplot (scores of samples and load factors of each variable) of the principal component analysis (PCA) (**A**), including the content of free amino groups (NH_2_), fructosamine (FRUCT), fluorescent AGE (FL-AGE), total AGE (T-AGE), and protein carbonyls (CARB), and dendrogram of hierarchical cluster analysis of the glycation inhibitory activity (**B**) of the samples of the BSA-GLU glycation system (21 days agitation, 37 °C, pH 7.4) BSA: negative control; GLU: BSA + glucose; AMG: BSA + glucose + aminoguanidine; CSE: BSA + glucose + CSE (12,5 mg mL^−1^); CGA1: BSA + glucose + 1 mmol L^−1^ CGA; CGA5: BSA + glucose + 5 mmol L^−1^ CGA; CGA10: BSA + glucose + 10 mmol L^−1^ CGA; GEN: BSA + glucose + 5 mmol L^−1^ genistein; MEL: BSA + glucose + 5 mmol L^−1^ melatonin.

PCA was carried out to find out the relationship between phytochemicals and antiglycative effects (Figure 3A). Six main principal components (PC) were obtained; the first two represented 76.3% of the total variability. PC1 separated the samples into two groups: samples treated with CSE or phytochemicals, along with BSA control, and samples treated with glucose and with aminoguanidine or CGA at the lowest concentration (1 mmol L^−1^). PC2 separated the samples into those treated with genistein or melatonin, and those treated with CGA (5 and 10 mmol L^−1^) and control (BSA). The sample treated with CSE presented intermediate values located between the two groups. For PC1, the content of total AGE and carbonyls were the dominant variables in the separation of samples treated with pure phytochemicals or CSE, and samples not treated or treated with aminoguanidine or 1 mmol L^−1^ CGA. PC2 correlated mainly with the antioxidant capacity (ABTS) of the samples. It is worth emphasising that the content in AGE, carbonyls, and free amino groups was negatively correlated with the antioxidant capacity. Figure 3B shows the dendrogram of the natural grouping of samples according to their antiglycative potential. As observed, the samples were organised into two groups, one formed by two subgroups, clustering the systems consisting of 1 mmol L^−1^ CGA and CSE (12.5 mg mL^−1^) and melatonin and aminoguanidine (both 5 mmol L^−1^), respectively. The second cluster grouped those samples with the highest antiglycative capacity, corresponding to the systems constituted by CGA (5 and 10 mmol L^−1^) and by genistein (5 mmol L^−1^).

Significant positive correlations (*r* ≥ 0.5; *p* < 0.01) were observed between the content of fluorescent AGE, total AGE, fructosamine, and protein carbonyls. Also, a correlation was observed between the concentration of total AGE, fructosamine, and carbonyls. The antioxidant capacity was negatively correlated (*p* < 0.05) with the free amino group content and carbonyl content. The inhibition of fructosamine and AGE formation (both fluorescent and total) negatively correlated with the inhibition of free amino groups’ loss (*r* ≥ ‒0.6; *p* < 0.01).

#### 3.2.2. MGO-Derivative AGE Formation in the BSA-MGO Model System

**Figure 4:**
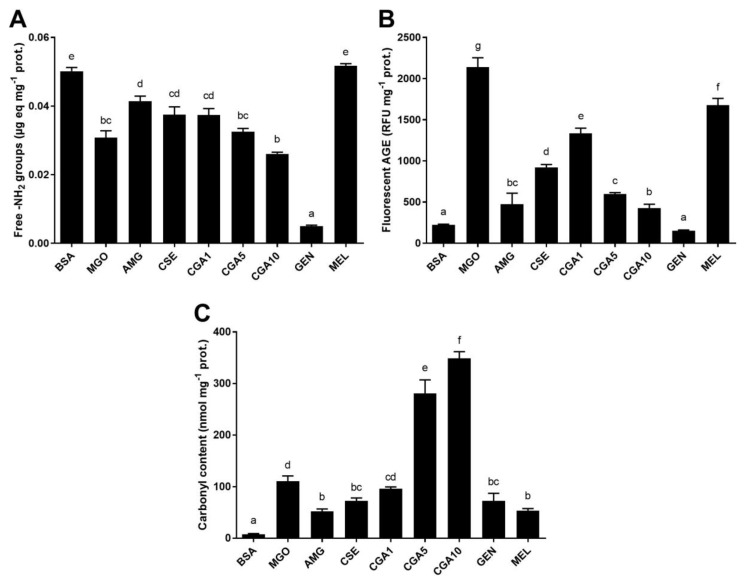
Contents of free amino groups (**A**), fluorescent AGE (**B**), and protein carbonyls (**C**) in the samples of the BSA-MGO (methylglyoxal) glycation system (72 h incubation, 37 °C, pH 7.4) BSA: negative control; MGO: BSA + methylglyoxal; AMG: BSA + methylglyoxal + aminoguanidine; CSE: BSA + methylglyoxal + CSE (12,5 mg mL^−1^); CGA1: BSA + methylglyoxal + 1 mmol L^−1^ CGA; CGA5: BSA + methylglyoxal + 5 mmol L^−1^ CGA; CGA10: BSA + methylglyoxal + 10 mmol L^−1^ CGA; GEN: BSA + methylglyoxal + 5 mmol L^−1^ genistein; MEL: BSA + methylglyoxal + 5 mmol L^−1^ melatonin. Results are presented as mean ± SD (*n* = 3). Bars with different letters denote significant differences (*p* < 0.05) when subjected to the Tukey multiple range test.

The results obtained on the formation of MGO-derivative AGE are shown in Figure 4. The content of free amino groups (Figure 4A) was significantly (*p* < 0.05) decreased (60%), suggesting the reaction of MGO with the protein. Only aminoguanidine and melatonin (5 mmol L^−1^) inhibited the loss of free amino groups significantly (*p* < 0.05). Treatment with genistein further decreased (90%) the content of free amino groups. It should be pointed out that the content of amino groups in CGA samples showed a dose–response effect, inversely proportional to CGA concentration (*r* = ‒0.999). The content of fluorescent AGE (Figure 4B) was significantly (*p* < 0.05) increased, approximately 10-fold in the sample treated with MGO. The phytochemicals significantly (*p* < 0.05) inhibited the formation of fluorescent AGE. The rate of inhibition was within 30–100%, with melatonin the less active and genistein the most effective compound. A strong linear correlation was observed between CGA concentration and the inhibition of the formation of fluorescent AGE (*r* = 0.917). Protein carbonyl content (Figure 4C) increased with MGO treatment. Aminoguanidine, genistein, melatonin, and CSE significantly (*p* < 0.05) inhibited protein oxidation. CSE and genistein inhibited 41%, while melatonin was more effective (59%). However, CGA seemed to promote such processes, with linearly correlated protein oxidation and CGA concentration (*r* = 0.948).

**Figure 5:**
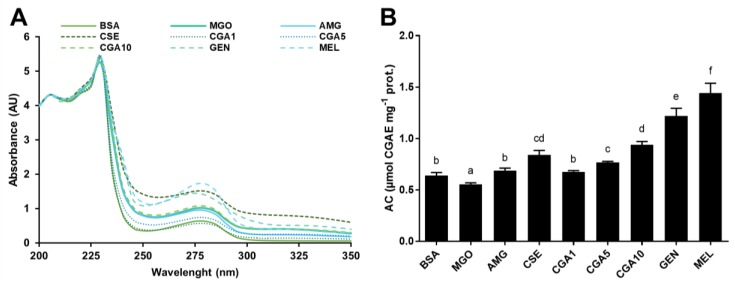
UV-Vis spectra (**A**) and antioxidant capacity (**B**) of the high molecular weight protein fractions (>30 kDa) isolated from the samples of the BSA-MGO glycation system (72 h agitation, 37 °C, pH 7.4) BSA: negative control; MGO: BSA + methylglyoxal; AMG: BSA + methylglyoxal + aminoguanidine; CSE: BSA + methylglyoxal + CSE (12,5 mg mL^−1^); CGA1: BSA + methylglyoxal + 1 mmol L^−1^ CGA; CGA5: BSA + methylglyoxal + 5 mmol L^−1^ CGA; CGA10: BSA + methylglyoxal + 10 mmol L^−1^ CGA; GEN: BSA + methylglyoxal + 5 mmol L^−1^ genistein; MEL: BSA + methylglyoxal + 5 mmol L^−1^ melatonin. Results are presented as mean ± SD (*n* = 3). Bars with different letters denote significant differences (*p* < 0.05) when subjected to Tukey multiple range test.

Figure 5 shows the modifications occurring in the HMW fraction of BSA treated with MGO in the presence or absence of the tested inhibitors. Structural modifications appeared not to be very noticeable for the treatments with aminoguanidine or 10 mmol L^−1^ CGA (Figure 5A). CGA at 1 and 5 mmol L^−1^ prevented the modifications caused by MGO. However, genistein and melatonin produced remarkable modifications in the native protein spectrum, with absorbance increased at 270–280 nm. The antioxidant capacity of the samples (Figure 5B), which indicates whether protein–phytochemical complexes were formed, demonstrated a significant (*p* < 0.05) change in the functionality of samples treated with CSE, CGA, genistein, and melatonin. The highest change in the antioxidant capacity of the proteins was observed in the genistein- and melatonin-containing samples. The CSE sample showed an antioxidant capacity significantly (*p* < 0.05) higher than that obtained for controls (BSA and MGO).

**Figure 6:**
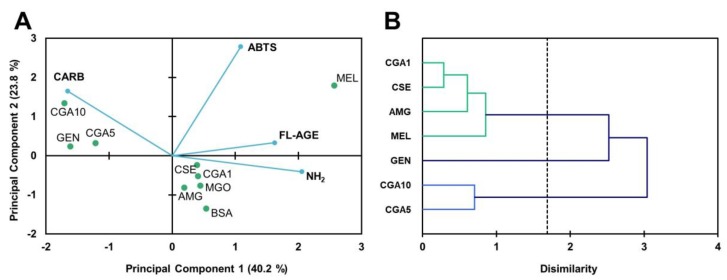
Biplot (scores of samples and load factors of each variable) of the principal component analysis (PCA) (**A**), including the content of free amino groups (NH_2_), fructosamine (FRUCT), fluorescent AGE (FL-AGE), total AGE (T-AGE), and protein carbonyls (CARB) (**A**), and a dendrogram of the hierarchical cluster analysis of the glycation inhibitory activity (**B**) of the samples of the BSA-MGO glycation system (72 h incubation, 37 °C, pH 7.4) BSA: negative control; MGO: BSA + methylglyoxal; AMG: BSA + methylglyoxal + aminoguanidine; CSE: BSA + methylglyoxal + CSE (12,5 mg mL^−1^); CGA1: BSA + methylglyoxal + 1 mmol L^−1^ CGA; CGA5: BSA + methylglyoxal + 5 mmol L^−1^ CGA; CGA10: BSA + methylglyoxal + 10 mmol L^−1^ CGA; GEN: BSA + methylglyoxal + 5 mmol L^−1^ genistein; MEL: BSA + methylglyoxal + 5 mmol L^−1^ melatonin.

The PCA (Figure 6A) established four components, representing the first two 69.8% of the variability. The PC1 divided the samples into two groups, depending on the content of free amino groups (variable of higher weight for this component), with those on the righ of high content, and those on the left of low content. PC2, in turn, divided the samples treated with genistein from those treated with CGA. The study of similarities found among samples, according to their percentages of inhibition for the analysed parameters, is illustrated in Figure 6B. The dendrogram showed the grouping of the inhibitors in two clusters. In the first, samples were sub-clustered: GEN (5 mmol L^−1^ genistein), on the one hand, and MEL (5 mmol L^−1^ melatonin), AMG (5 mmol L^‒^1 aminoguanidine), CSE (12.5 mg mL^−1^), and CGA1 (5 mmol L^−1^ CGA) on the other. In the second cluster, samples with CGA (5 and 10 mmol L^−1^) were found. Samples with CGA at concentrations of 5 and 10 mmol L^−1^ (the highest concentrations) were separated by their inducing effect on the formation of protein carbonyls. The samples with genistein were separated, showing that this had compound the highest percentages of inhibition. CSE and AMG were grouped; their effects on the inhibition of glycoxidation reaction products formations were very similar. On the other hand, significant negative correlations (*p* < 0.01) were observed between the percentage of the inhibition of fluorescent AGE formation and the inhibition of amino group loss (*r* = ‒0.778).

### 3.3. Formation of Protein–Phytochemical Complexes

To further confirm the formation of BSA–phytochemical complexes as the mechanism of action of CGA, genistein, and melatonin we evaluated the formation of complexes by fluorescence quenching and using molecular docking to investigate *in silico* how the phytochemicals bound to the protein.

#### 3.3.1. Fluorescence Spectra

**Figure 7:**
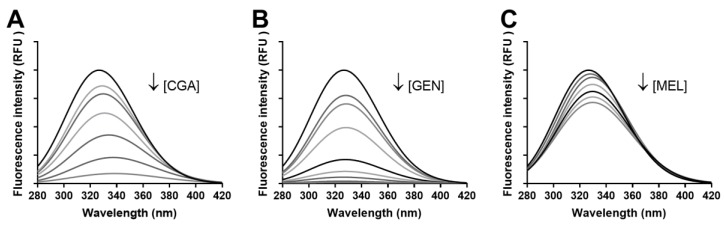
Fluorescence spectra of BSA showing fluorescence quenching in the absence and presence of varying concentrations of CGA (5–200 µmol L^−1^) (**A**), genistein (5–1000 µmol L^−1^) (**B**), and melatonin (5–200 µmol L^−1^) (**C**). λ_ex_ = 250 nm, pH 7.4, T = 310 K.

Fluorescence spectra for BSA-CGA, -GEN, and -MEL are depicted in Figure 7. Experiments carried out with glucose, MGO, and aminoguanidine demonstrated that these compounds did not complex with the protein (at the assayed concentrations) nor modify its fluorescence spectra. CGA and genistein highly quenched fluorescence, even at the lowest concentration tested (5 µmol L^−1^). 

The fluorescence intensity of BSA gradually diminished while increasing the concentration of the compound. Therefore, these results would confirm the formation of different strength protein–phytochemical complexes for the three bioactive compounds evaluated in this work. Since the compounds bound the protein differently, we calculated quenching and binding parameters of the phytochemicals (Table 2). For the fluorescence quenching measurement, the decrease in intensity is usually described by the well-known Stern–Volmer equation. In the linear range of the Stern–Volmer regression curve, the average *K_SV_* and *k_q_* were determined. Values of *k_q_* higher than the maximum value possible for diffusion-limited quenching in water (∼1010 M^−^^1^ s^−^^1^) indicate an interaction among the compounds and BSA. Here, the *k_q_* values were in the range of 10^12^ to 10^13^, which confirmed the presence of a specific interaction occurring between CGA, genistein, and melatonin and BSA. Besides this, the binding constant value (*K_a_*) and the number of binding sites (*n*) were obtained from the double-logarithm curve. The *K_a_* values suggest the tested phytochemical showed a moderate affinity to BSA since the documented *K_a_* values of non-covalent association of BSA with drugs are mostly in the range of 10^4^–10^6^ M^−1^.

#### 3.3.2. In Silico Prediction of Protein–Phytochemical Interactions

**Figure 8:**
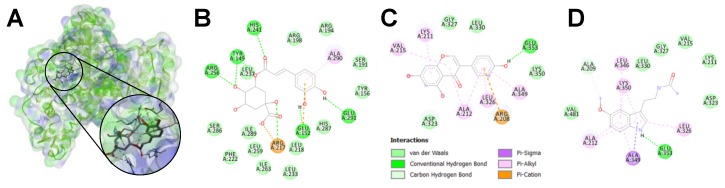
Representative molecular interaction of CGA with BSA (**A**), and 2D diagrams of the interaction of BSA with chlorogenic acid (**B**), genistein (**C**), and melatonin (**D**) determined using *in silico* molecular docking. The figure shows the compounds bound to the IIA domain of BSA and the interacting residues in each complex.

Additionally, molecular docking studies illustrated the binding energies and the potential interaction of the compounds with the aminoacidic residues of BSA (Table 3, Figure 8). CGA, genistein, and melatonin exhibited strong binding energies (from ‒5.5 to ‒9.0 kcal mol^−1^), demonstrating the occurrence of protein complex formation. As observed in the fluorescence spectra, the interaction of glucose, MGO, and aminoguanidine with BSA is weaker than that of CGA, genistein, and melatonin. 

As observed, CGA, genistein, and melatonin could block a great number of lysine and arginine residues (BSA amino groups), the main reactants for the Maillard reaction. In most of the observed interactions, the phytochemicals would interact with the same residues that glucose would. Additionally, these three bioactive compounds might protect other lysine and arginine amino residues. Therefore, the formation of protein–phytochemical complexes was proven, both with fluorescence spectra and the molecular docking experiments. 

## 4. Discussion

In the present work, the potential to inhibit the Maillard reaction of other CSE components besides CGA, such as isoflavones and melatonin, was evaluated for the first time. The results indicate that CSE is a sustainable source of phytochemicals, exhibiting higher TPC content than previously described [11]. The content of CGA and caffeine was higher in the present CSE than those values previously reported. The differences found in the concentrations of these bioactive compounds may be due to multiple factors, such as agronomic, geographical origin, species, variety, processing conditions (wet or dry), and roasting degree [29,30]. In the present research a mixture of Arabica and Robusta coffee silverskin (50:50 *w*/*w*), the most commercially available coffee species worldwide, was employed for obtaining CSE. This blend could better represent the coffee market and balance the differences in phytochemicals among the two varieties [31,32]. The melatonin content was higher than that present in the coffee drink [33,34] and several orders of magnitude higher than in other foods considered rich in melatonin (nuts, cherries, or grapes, among others) [35]. The studied extract showed higher antioxidant capacity than other extracts from coffee by-products or extracts of coffee silverskin reported by others [14]. 

The reaction between the carbonyl group of a reducing sugar and a free amino group of a protein triggers a series of subsequent reactions, giving rise to fructosamine, carbonyls structures, and AGE. This study provides information about the influence of phytochemicals which compose CSE on the inhibition of different stages of Maillard reaction. The *in vitro* reaction models selected, BSA-GLU and BSA-MGO, generate Maillard reaction products of different nature. The BSA-GLU system showed the formation of early and advanced Maillard reaction products. In contrast, The BSA-MGO model provided useful information on the formation of AGEs and oxidation/glycoxidation products. 

CSE and pure CGA, genistein, and melatonin inhibited the Maillard reaction under our particular experimental conditions. Pure phenolic compounds decreased the free amino groups’ content, suggesting an inhibition of the Maillard reaction at its very early stages. Phenolic compounds can block amino groups when bound to the protein, inhibiting the formation of Amadori products and preventing glycation [36]. Genistein and melatonin were less effective inhibitors of the early stages of the Maillard reaction than CGA. However, they inhibited the formation of protein carbonyls through glycoxidation. CGA inhibited the formation of fructosamine, indicating that hydroxycinnamic acids have different antiglycative mechanisms than genistein and melatonin. The ability of CSE and its components to inhibit the formation of fluorescent and total AGE (glucose model system), at the tested concentrations, was higher than that of aminoguanidine. The antiglycative capacity of the extract seems not to be due solely to CGA, since, at similar concentrations of CGA, CSE has a higher inhibitory potential [14]. Other compounds present in CSE have been reported as potential inhibitors of the advanced glycation of proteins [14]. Among them, melanoidins have been suggested to inhibit AGE formation, acting as radical scavengers and inhibiting dicarbonyl reactive compound formation during glucose autoxidation [37,38]. Likewise, other antioxidant compounds, mainly phenolics and natural extracts, have also exhibited similar effects [39,40,41]. The measurement of AGE through ELISA techniques, however, entails some limitations (poor precision and susceptibility to matrix effects); the actual concept of AGE should be validated using HPLC-MS/MS techniques [42]. Carbonyl content measurement showed the protective effect of CSE, genistein, and melatonin against protein oxidation. Protein carbonylation is an index of the oxidation of amino groups in proteins, usually caused by oxidative stress or reaction with reducing sugars (glycation or glycoxidation). Previous studies have shown correlations among the levels of fluorescent AGE, N^ε^-(carboxymethyl)lysine (CML), and N^ε^-(carboxyethyl)lysine (CEL) with the levels of protein carbonyls [43,44]. CGA induced protein carbonyl formation. It is known that some CGA derivatives, such as oxidation products and isomers, may be able to act as substrates and precursors of the Maillard reaction and polymerisation reactions, which would explain the formation of carbonyls in the samples containing 5 and 10 mmol L^−1^ CGA (BSA-MGO system) [45]. In addition, CGA has been shown to exert pro-oxidant effects, apart from antioxidant effects. In oxidative conditions, such as the presence of MGO, a phenoxyl radical may be produced from CGA, reacting with proteins and oxidising them [46]. 

The direct protein–phytochemical interaction creates competition for free amino groups, an effect that decreases the binding of reactive carbonyls to protein, and therefore decreases AGE formation. At the same time, this loss of free amino groups is associated with a new functionality of protein, presenting a higher antioxidant capacity than the native one. Protein modifications indicated in this study may help to understand the mechanism of the action of antioxidants on protein glycation. The increased absorbance (270–280 nm) in the samples that were treated with genistein and melatonin, due to the formation of HMW protein–phytochemical complexes, correlated with the increased antioxidant capacity of the protein. Thus, the change in protein structure was associated with the change in protein function. Likewise, the fluorescence quenching and the *in silico* predicted interactions of CGA, genistein, and melatonin seemed to prove the formation of these protein–phytochemical complexes and exposed the strength and nature of the interactions. Previous studies using spectroscopic and docking techniques demonstrated that chlorogenic acid was able to interact with human serum albumin, and in a different manner depending on the isomer (chlorogenic acid, neochlorogenic acid, or cryptochlorogenic acid) [47]. Likewise, genistein interaction with BSA was assessed in another study, exhibiting similar quenching and binding parameters, that were improved when genistein was derivatised with peptides [48]. The protection of lysine and arginine amino residues from the reaction with the reactive carbonyl group of sugars or dicarbonyls was observed for both the phenolics (CGA and genistein), but also melatonin. Phenols bind to highly nucleophilic thiols, amino groups, and hydrophobic aromatic groups of proteins [49]. Three possible types of non-covalent interactions between proteins and phenolics have been proposed: hydrogen bonds, hydrophobic interactions, and ionic bonds [50]. Here, we observed them *in silico*, as well as carbon–hydrogen bonds, π-interactions, or salt bridges. Proteomic studies with Matrix-Assisted Laser Desorption/Ionization-Time Of Flight (MALDI-TOF) have determined which are the binding sites of CGA and its metabolites that, in turn, inhibit MGO binding [36]. CGA had higher reactivity than MGO, inhibiting the formation of most protein modification in both lysine and arginine residues [51].

**Figure 9:**
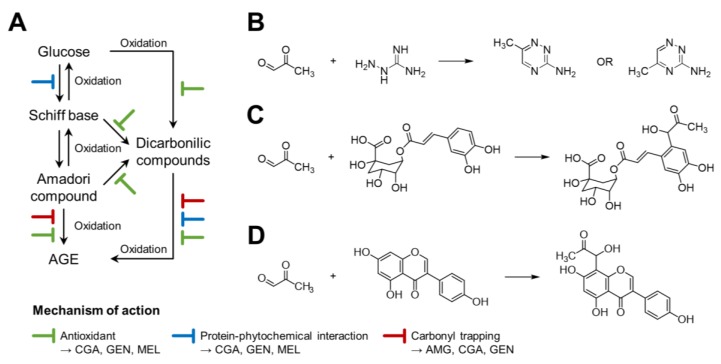
Illustrative diagram of the inhibitory mechanism (**A**) of coffee silverskin extract and the compounds (aminoguanidine, chlorogenic acid, genistein, and melatonin) studied in this work and the reactions of methylglyoxal trapping by aminoguanidine (**B**), chlorogenic acid (**C**), and genistein (**D**).

The antiglycative ability of the studied compounds, however, was not due exclusively to their protein binding properties. The ability to inhibit AGE formation of these compounds may be associated with their antioxidant activity, their ability to chelate metal ions, to trap carbonyl radicals, or to inhibit protein-cross-linking activity [52,53,54]. Since most of the reactions comprised in the Maillard reaction (Figure 9A) include oxidation processes, the direct antioxidant effect of bioactive compounds may be one of the major ways of decreasing the production rate of Maillard reaction products and the formation of AGE. 

As shown, aminoguanidine does not interfere with the formation of early Maillard reaction products (Amadori compounds) but inhibits subsequent rearrangements which are essential for crosslinking reactions, working as a post-Amadori reactive carbonyls quencher (Figure 9B) [55,56]. We demonstrated that, conversely to the other studied compounds, aminoguanidine cannot bind the protein nor form BSA–aminoguanidine complexes. 

The results suggest that the antiglycative capacity of CGA may be due to several mechanisms (Figure 9A,C), including its antioxidant character, inhibiting fructosamine degradation, as well as its binding to BSA, inhibiting the formation of fructosamine and AGE, and to its MGO trapping capacity (Figure 9C) [14]. CGA could inhibit the formation of fructosamine. CGA, at the concentrations found in CSE, may play a major role in inhibiting the Maillard reaction.

The results show that the binding capacity of genistein to proteins, with the consequent change in structure-function, decreases the formation of AGE. It was also reported that genistein inhibits the degradation of Amadori products, inhibiting the formation of carbonyls (and consequent protein oxidation) and the formation of AGE. Genistein would also have the ability to form adducts with MGO (Figure 9D) [15,16]. Genistein and other isoflavones that are in lower concentration than CGA may display a minor, but significant contribution to the total antiglycative capacity of CSE.

Melatonin, a compound with a non-phenolic structure, showed a primarily antioxidant behaviour, and it is also able to join to proteins, modifying their structure and functionality. The results seem to indicate the interaction protein melatonin is less favoured than that for other studied phytochemicals, and this may be due to other chemical reactions different to the Maillard reaction. Melatonin inhibited the formation of carbonyl compounds associated with protein oxidation and AGE formation. 

CSE inhibited fructosamine and AGE formation, as well as protein oxidation. CSE antiglycative properties can be associated with the synergic effect of several phytochemicals, including CGA, isoflavones, and melatonin. CSE inhibited the Maillard reaction by all the mechanisms ascribed to the pure phytochemicals. In addition, melanoidins may also contribute to the observed effect [13]. Further studies for estimating the individual contribution of phytochemicals and compounds formed during roasting (melanoidins) to the antiglycative capacity of CSE should be conducted. However, the results derived from the present research seem to indicate that phytochemicals play a fundamental role as antiglycative agents of the extract. 

The inhibitors of AGE production are considered targets in the management of diabetes and hyperglycaemia complications [57]. The search for natural alternatives to aminoguanidine and other antiglycative drugs with adverse side effects is of increasing interest. The use of natural extracts obtained from plants and food by-products can be a sustainable alternative to the use of synthetic drugs. 

## 5. Conclusions

We described, for the first time, the potential role of other phytochemicals, besides CGA, that compose CSE, such as genistein and melatonin, as contributors of its antiglycative character. These bioactive compounds have demonstrated the ability to inhibit different steps of the Maillard reaction through different mechanisms. Future studies at other levels of experimentation should be conducted to demonstrate the potential of CSE to decrease the complications of diabetes and oxidative stress-related diseases by inhibiting different steps of the Maillard reaction.

## Figures and Tables

**Table 1:** Phytochemical characterisation of the coffee silverskin extract (CSE), including total phenolic compounds (TPC), chlorogenic acid (CGA), total flavonoid content (TFC), melatonin, caffeine, and antioxidant capacity measured by the ABTS^•+^ method.

Phytochemicals	Concentration (mean ± SD)
TPC (μg CGAE mg^−1^ extract)	70.4 ± 4.2
CGA (μg mg^−1^ extract)	35.1± 0.7
TFC (μg RE mg^−1^ extract)	83.2 ± 4.3
Melatonin (ng mg^−1^ extract)	2.0 ± 0.1
Caffeine (μg mg^−1^ extract)	47.0 ± 0.5
ABTS^•+^ (μg CGAE mg^−1^ extract)	166.5 ± 1.8

**Table 2:** Experimentally determined quenching and binding parameters for BSA–phytochemical complexes obtained at equal conditions of pH (7.4) and temperature (37 °C/310 K), including the Stern−Volmer quenching constant (*K_SV_*), bimolecular quenching constant (*k_q_*), binding constant (*K_a_*), and the number of binding sites (*n*).

Compound	*K_SV_* (10^4^ mol L^−1^)	*k_q_* (mol L^−1^ s^−1^)	*K_a_* (mol L^−1^)	*n*
Chlorogenic acid	4.42 ± 0.14	(7.64 ± 0.24) × 10^12^	4.78 ± 0.19	1.05 ± 0.04
Genistein	9.52 ± 0.10	(1.65 ± 0.18) × 10^13^	5.36 ± 0.12	1.12 ± 0.03
Melatonin	0.70 ± 0.02	(1.21 ± 0.34) × 10^12^	3.11 ± 0.18	0.82 ± 0.04

**Table 3:** Binding energies (kcal mol^−1^) between BSA (six different domains) and the pro-glycation (glucose and MGO) and antiglycative (aminoguanidine, CGA, genistein, and melatonin) agents predicted using *in silico* molecular docking and the interacting arginine and lysine residues.

BSA Domain	GLU	MGO	AMG	CGA	GEN	MEL	Interacting Amino Acids *
IA	‒4.6	‒3.7	‒3.6	‒6.1	‒6.6	‒5.8	Lys^4^, Arg^10^, **Lys^20^**, Lys^41^, **Lys^131^**, **Lys^132^**
IB	‒5.9	‒3.8	‒4.0	‒8.2	‒8.7	‒6.7	Arg^144^, Arg^194^, Arg^196^, Arg^198^, Arg^256^, Lys^431^, Arg^458^
IIA	‒5.9	‒3.8	‒4.1	‒9.0	‒8.4	‒6.8	**Arg^194^**, **Arg^198^,** Arg^208^, Lys^211^ **Arg^256^**, Lys^350^, Arg^347^, Arg^484^
IIB	‒5.4	‒3.3	‒4.0	‒7.9	‒8.4	‒6.9	**Arg^208^**, Lys^211^, **Lys^350^**, Arg^347^, Arg^483^, Arg^484^
IIIA	‒5.4	‒3.5	‒4.1	‒8.1	‒8.0	‒7.5	Arg^217^, Arg^347^, Arg^409^, Lys^413^, Lys^431^, Arg^458^, **Arg^484^**
IIIB	‒4.8	‒3.4	‒3.6	‒6.2	‒6.2	‒5.5	Arg^427^, Arg^431^, **Lys^499^**, Lys^524^, **Lys^533^** Lys^556^, Lys^573^

* Amino acids in bold interacted both with glucose and the studied compounds (CGA, genistein, or melatonin).
